# Application of ultrasound molecular imaging based on compressed sensing reconstruction algorithm to phase change drug-loaded PLGA nanoparticles targeting breast cancer MCF-7 Cells

**DOI:** 10.12669/pjms.37.6-WIT.4852

**Published:** 2021

**Authors:** Yufeng You, Wusong Cheng, Hongbo Chen

**Affiliations:** 1 Yufeng You, Master of Medicine. Department of Radiology, The Central Hospital of Enshi Tujia and Miao Autonomous Prefecture, Enshi, 445000, Hubei, China; 2 Wusong Cheng, Master of Medicine. Department of Radiology, The Central Hospital of Enshi Tujia and Miao Autonomous Prefecture, Enshi, 445000, Hubei, China; 3 Hongbo Chen, Master of Medicine. Department of Radiology, The Central Hospital of Enshi Tujia and Miao Autonomous Prefecture, Enshi, 445000, Hubei, China

**Keywords:** Ultrasound molecular imaging, Compressed sensing reconstruction algorithm, PLGA nanoparticles, Breast cancer, MCF-7

## Abstract

**Objectives:**

To study the ability of aptamer-modified nano-gold rods and liquid carbon-targeted PLGA nanoparticles to target in vitro using compressed sensing reconstruction algorithm, and observe the phenomenon of mediating ultrasound / photoacoustic imaging.

**Methods:**

PLGA nanoparticles were prepared by a double emulsification method, and the MUC1 aptamer was connected to the PLGA nanoparticles by the carbodiimide method to obtain an “aptamer-PLGA nanoparticle” targeted phase change contrast agent. Fluorescence microscopy was used to detect the in vitro targeting of breast cancer MCF-7 cells specifically identified by it, and three control groups were set up: the ordinary nanoparticle group, the aptamer interference group, and the HELA cell group. A photoacoustic instrument was used to observe the phenomenon of enhanced ultrasound / photoacoustic signal mediated in vitro.

**Results:**

Many targeted nanoparticles were clustered around MCF-7 cells and bound firmly, but no specific binding was observed in the non-targeted nanoparticles group, the aptamer interference group and the HELA cell group. After the targeted nanoparticle was excited by the photoacoustic instrument, the ultrasonic signal and the photoacoustic signal were significantly enhanced compared with before the excitation.

**Conclusion:**

The successfully prepared targeting nanoparticles have good targeting and specificity for breast cancer MCF-7 cells, and it has obvious effects on ultrasound / photoacoustic imaging, and has the potential to become a dual-mode ultrasound / photoacoustic targeted contrast agent. The various characteristics provide experimental basis for subsequent in vivo targeting experiments and are expected to become good target diagnostic molecular probes.

## INTRODUCTION

Breast cancer is a common oncological disease among women all over the world, and 42% of female cancer cases worldwide occur in developing countries.^1^,^2^ Breast cancer and tumors are seriously affecting people’s lives. Early image diagnosis plays a vital role in cancer diagnosis.^3^,^4^ With the rapid development of molecular imaging technology, molecular-level imaging has laid a solid foundation for early diagnosis and treatment of various diseases.^5^,^6^ Targeted multi-modal contrast agents have attracted much attention because they can be used in multiple imaging modes to obtain relatively complete biological information and highly specific recognition of targets.^7^,^8^ Photoacoustic imaging is a new type of non-destructive medical imaging mode based on photoacoustic effects.^9^,^10^ It combines the advantages of pure optical imaging with high contrast and ultrasonic imaging with high penetration depth. It has been under discussion in recent years.^11^-^13^

In this study, a new type of targeted molecular probe, an aptamer, was connected to polymer nanoparticles coated with liquid carbon and superior optical contrast agent nano-gold rods to construct a targeted ultrasound / photoacoustic dual mode State contrast agent, and explore its targeting ability and photoacoustic / ultrasonic imaging effect in vitro.

## METHODS

### Experimental materials and instruments

Experimental materials: carboxyl-terminated lactic acid-light acetic acid copolymer (PLGA-COOH, polymerization ratio 50:50); nano-gold rod (GNR); perfluoro hexane (PFH); chloroform; polyvinyl alcohol (PVA); 1,2-dichloroethane (EDC); N light succinimide (NHS); MUC-1 aptamer (nucleic acid sequence 5’-NH2-GCAG-TTGATCCTTTGGATA CCCTGG-3’); TE Buffer; DEPC water; Dio fluorescent dye; Dli fluorescent dye; 1640 culture medium.

### Experimental instruments:

electronic balances; acoustic vibrators; high-speed dispersing homogenizers; magnetic stirrers; centrifuges; constant temperature shakers; inverted fluorescence microscopes; photoacoustic instruments.

### Experimental methods:

Preparation of aptamer-PLGA nanoparticle-targeted contrast agent: (1) Preparation of PLGA nanoparticles coated with nano-gold rods and PFH: a mixture containing 200µl of PFH and 1 ml of nano-gold rods was shaken with a vibrator 1 A purple emulsion was obtained after few minutes. Take 50mg of PLGA and dissolve it in 2ml of dichloromethane. After the solution is completely dissolved, add the above purple emulsion to it, continue the sonication for 1min, and then add 15ml of PVA to it, and homogenize with a homogenizer. After three minutes, magnetically stir two to four ratios to completely evaporate the dichloromethane. Finally, after centrifugal washing with double-distilled water for several times, the precipitate was dispersed in 2 ml of double-distilled water to obtain PLGA nanoparticles (CGNP) wrapped with nano-gold rods and liquid frequency carbon, and stored at 4°C.

Linking of aptamer with PLGA nanoparticles: Take 50µl (10mg/ml) of PLGA nanoparticles, and add 400mmol/L 1,2-dichloroethane (EDC) and 100mmol/L N -200 μl each of light succinimide (NHS). After shaking and incubating at room temperature for 30 minutes, centrifugation and washing with DEPC water several times to remove unreacted EDC and NHS, to obtain fluorenyl-activated PLGA nanoparticles. After centrifuging the 1 OD MUC-1 aptamer, add 40 µl of TE buffer solution to dissolve it, and then place it at 85 ° C in a constant temperature water bath for 10 minutes, then quickly cool it in ice water for 10 minutes. Add “denatured-renatured” aptamers to PLGA nanoparticles after fluorenyl activation, and incubate for two hour at room temperature with shaking. Centrifugation and washing with DEPC water for several times to remove the free aptamers, to obtain “aptamer-PLGA” targeted nanoparticles (AP-GNP).

General properties of targeted nanoparticles. After the AP-GNP was appropriately diluted with double-distilled water, its morphology and distribution were observed with an optical microscope, and its particle size was detected with a Malvern particle size analyzer.

Targeting Nanoparticles for in vitro targeting. Breast cancer MCF-7 cells and cervical cancer HELA cells were cultured in a complete medium with a mass ratio of 1:10 of fetal bovine serum to 1640 medium at 37 ° C, volume fraction 5% CO2, and complete humidity. MCF-7 cells and HELA cells in logarithmic growth phase were seeded in 35 mm cell culture dishes at 1×105 cells/well, and cultured for 24 hour. The experiment is divided into four groups, three groups are MCF-7 cells, one group is HELA cells, and each group has 3 petri dishes. The AP-GNP and GNP used are labeled with Dli: the heart AP-GNP group, and each MCF- 7 Add AP-GNP50ul to the cell culture dish; 1. GNP group, add GNP 50µl to each MCF-7 cell culture dish; 2. aptamer interference group, add the adapter to each MCF-7 dish first (AP) 8μl (1μg/μl) after incubating for two hours, then add AP-GNP50μl to the petri dish; 3. HELA cell control group, add human AP-GNP 50μl to each HELA cell petri dish. After incubation for the above four groups, continue to incubate for 2 h, and then repeatedly wash the culture dish with PBS solution to remove the nanoparticles that have not specifically bound to the cells. Then add 20 μl of Dio fluorescent dye (1 mg / ml) to each culture dish. The cells were stained. After 10 minutes, the culture dish was repeatedly washed with PBS solution again, and the binding of the four groups of nanoparticles to the cells was observed under an inverted fluorescence microscope.

Detection of near-infrared absorption peaks of nanoparticles. An appropriate amount of AP-GNP was placed in the gel cavity model, and the AP-GNP was irradiated in a wavelength range of 680×900 mm with a laser to obtain the change curve of its photoacoustic signal intensity under different wavelengths of irradiation.

Targeted nanoparticle-mediated in vitro ultrasound and photoacoustic imaging. Photoacoustic imaging was performed using the measured wavelengths of the near-infrared absorption peaks of the targeted nanoparticles. The experiments were divided into three groups: 1. aptamer-modified nanoparticle group coated with nano-gold rods and liquid jaw carbon (AP-GNP group); 2. blank nanoparticle group coated with liquid-locked carbon (NP group); Double distilled water group. AP-GNP was placed in the gel cavity model, and the ultrasound mode of the photoacoustic instrument was used to acquire the ultrasound image and the contrast mode image before the laser irradiation, and then the photoacoustic signal was used to collect the photoacoustic signal. After completion, the laser was continued to be irradiated for two minutes, and the ultrasound image and the contrast mode image were acquired again in the ultrasound mode. The NP group and double-distilled water group used the same processing method as the AP-GNP group to obtain ultrasound signals and photoacoustic signals.

### Compressed sensing reconstruction algorithm

Compressed sensing is a new information acquisition theory. It is a method of signal acquisition and reconstruction based on sparse representation of signals, non-correlation of measurement matrices, and approximation theory. Let x be a signal of length N, x is sparse in the transformation domain Ψ, i.e.


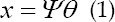


Where Ψ is the sparse transform basis.


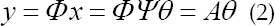


The solution with the sparse structure can be found through the l0 norm optimization problem:


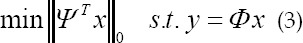


Since the optimization problem of equation (3) is a difficult NP-hard problem, the l¬1 constraint can be used instead of the l¬0 constraint:


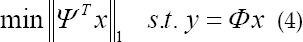


For a vector of length N (actually refers to an N-dimensional discrete outlier signal), only K of its N element values are non-zero, where K << N, then we call this vector K sparse or strictly K-sparse; in practice, it is not easy to achieve strict K-sparseness. Generally, if the values other than the K values are small, we consider the vector to be sparse.

The test signals are sparsely decomposed with different sparse bases, and a threshold is set. Coefficients smaller than the threshold are regarded as 0, and the sparseness of the signals under each sparse base is compared. Common sparse bases are discrete Fourier basis (FFT), discrete cosine transform basis (DCT), discrete sine transform basis (DST), discrete Hartley transform (DHT), and discrete W transform. The test signal (signal length N=1,841) is obtained in Table-I. The test signal (signal length N=300) is obtained in Table-II.

**Table-I:** Sparsity of test signals under different sparse basis.

	*FFT*	*DCT*	*DST*	*DHT*	*W*
c=0.01	1313	1468	1657	1473	1477
c=0.05	311	490	1107	494	487
c=0.1	183	220	945	218	221

**Table-II:** Sparsity of test signals under different sparse basis.

	*FFT*	*DCT*	*DST*	*DHT*	*W*
c=0.01	230	247	275	250	249
c=0.05	51	77	200	103	98
c=0.1	33	38	170	46	45

## RESULTS

### General properties of targeted nanoparticles

**Fig.1:**
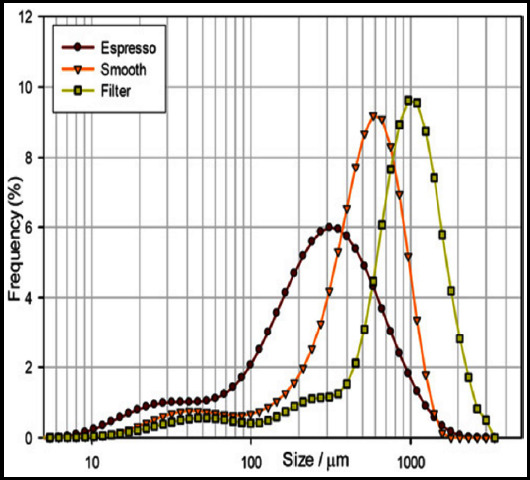
Target particle size distribution measured by Malvern particle size analyzer.

Under the light microscope, it was observed that the nanoparticles were spherical, the size was uniform, the dispersion was good, and there was no obvious aggregation and adhesion. The average particle size of the targeted nanoparticles measured by the particle size analyzer was (362.40 ± 31.13) nm (Fig.1).

Inverted fluorescence microscopy showed that both the MCF-7 cells labeled with Dia and HELA cells were green, and the AP-GNP and GNP labeled with Dli were red. The AP-GNP group was visible under the fusion channel. Targeting nanoparticles and cell specificity Most of the surface of the cell membrane were combined, and a small number of targeted nanoparticles were swallowed into the cell; no obvious specific binding between nanoparticles and cells was observed in the GNP group, aptamer interference group, and HELA cell group.

In the AP-GNP group, the echo intensity of ultrasound and contrast modes increased significantly before and after laser irradiation, but no significant changes were observed in the NP group and the double-distilled water group. Apparent photoacoustic signals were detected in the AP-GNP group, but no photoacoustic signals were detected in the NP and double-distilled water groups.

## DISCUSSION

With the rapid development of nanomedicine technology, ultrasound contrast agents have developed from micrometers to nanometers, showing strong penetrating power. The representative PFC NP is temperature-free and non-toxic due to its chemical properties.^14^,^15^ It can reach tumor cells in the vascular endothelial space and undergo a liquid-to-gas phase transition to enhance the ultrasound imaging, so it has been widely used in ultrasound molecular imaging.^16^-^19^ You et al.^20^ Prepared a multifunctional Fe3O4-PFH/PLGA nanoparticle, which undergoes a phase change in the PFH core of highly focused ultrasound and can work in synergy with high-intensity focused ultrasound to effectively improve the therapeutic effect of tumors. In this study, in vitro targeting experiments showed that many nanoparticles labeled with red fluorescence in the AP-GNP group were tightly attached to the cell membrane surface of MCF-7 labeled with green fluorescence, and some nanoparticles were swallowed. In the GNP group, the nanoparticles did not specifically bind to the cells; while in the aptamer interference group, simple aptamers blocked the binding sites of the cells, resulting in no obvious specific binding between the nanoparticles and the cells. Although AP-GNP nanoparticles and HELA cells in the HELA cell group did not show obvious specific binding, there were still very few nanoparticles adhered to the cell membrane. It may be caused by electrostatic adsorption, which can fully prove the high specificity of AP-GNP to the target.

Foreign scholar Rapoport et al.^21^ used high molecular polymer polylactic acid-polyethylene glycol block copolymer and polyethylene glycol-b-polyglycolide to wrap perfluoropentane to prepare the doxorubicin-loaded micelles and drug-loaded nanoparticles; it was found that doxorubicin was released quickly under certain energy ultrasound irradiation and could be successfully taken up by breast cancer cells, clarifying the drug release mechanism and finding that ultrasound enhances the drug release of target cells. Ultrasound imaging experiments in this study showed that the ultrasound echo intensity and contrast pattern of the AP-GNP group were greatly enhanced before and after the laser irradiation, while the echoes of the NP group and the double distilled water group had no significant changes. Laser irradiation showed that nano-packages were gold rods, and liquid nanoparticles targeting carbon had successfully undergone a liquid-gas phase change, which provided a strong experimental basis for future in-vivo ultrasound enhancement experiments.

## CONCLUSIONS

In this experiment, aptamer-modified nano-gold rods coated with polymer and liquid carbon vibrating polymer-targeted bi-modal nanoparticles were successfully prepared. There in vitro targeting ability is good, and the ultrasound and photoacoustic imaging effects are obvious. Combining the high imaging depth of ultrasound imaging with the high resolution of photoacoustic imaging, coupled with its good targeting performance, it has become a contrast agent with great practical value.

### Authors’ Contribution:

**YY:** Conceived the study, literature review, data analysis and drafting the manuscript.

**WC:** Helped in design, data collection, article drafting & critical revision.

**HC:** Takes the responsibility and is accountable for all aspects of the work in ensuring that questions related to the accuracy or integrity of any part of the work are appropriately investigated and resolved.
